# Usage and appraisal of educational media by homeopathic therapists – A cross sectional survey

**DOI:** 10.1186/1472-6882-12-95

**Published:** 2012-07-13

**Authors:** Max Escher, Horst Christian Vollmar, Andreas Holling, Christa Raak, Thomas Ostermann

**Affiliations:** 1 Center for Integrative Medicine, Witten/Herdecke University, Gerhard Kienle-Weg 4, 58313, Herdecke, Germany; 2 Department of General Practice, University of Düsseldorf, Moorenstraße 5, 40225, Düsseldorf, Germany; 3 Institute of General Practice and Family Medicine, Witten/Herdecke University, Alfred-Herrhausen-Str. 50, 58448, Witten, Germany; 4 Bönninghausen Institute for Holistic Healing, Maximilianstr. 15A, 48147, Münster, Germany

**Keywords:** Educational media, Homeopathy, Primary care, Continuing medical education, Knowledge translation

## Abstract

**Background:**

During recent years the market for homeopathic education media has increasingly diversified with old (books, seminars) and new media (video-seminars, pc-programs, homeo-wiki and internet-courses). However, little is known about homeopaths’ preferences in using educational media and their requirements of this topic.

**Aim:**

This survey was designed to gain a better understanding of the usage and appraisal of educational media by homeopaths.

**Methods:**

192 homeopathic practitioners (GPs and health practitioners) at a educational conference were asked to answer a standardized questionnaire covering the topics “formal education and context of work” (9 items), “homeopathic practise and usage (24 items), “utilization of educational media” (9 items) and “favoured attributes for educational media” (11 items).

**Results:**

Out of 192 homeopaths who attended the conference, 118 completed the questionnaire (response rate 61.5%). For their continuing homeopathic education they predominantly indicated to use books (scale value from 0 = never to 2 = always: 1.72) and seminars (1.54) whereas journals (0.98) and the internet (0.65) were used less often. The most favoured attributes concerning medical education media were reliability (1.76), relevance for clinical practice (1.74) and user friendliness (1.6). Less favoured attributes were inexpensiveness (1.1), graphical material (0.92) and interactivity (0.88).

**Conclusions:**

The survey illustrates the current situation of medical education media in homeopathy. Although there are parallels to earlier research conducted in conventional GPs, homeopaths are more likely to refer to classical media. New education tools should be designed according to these preferences.

## Background

The translation of knowledge into practice is essential in order to maintain and increase the quality of daily practice [1]. Thus, lifelong learning is a key issue for health care professionals in keeping up-to-date with new medical knowledge and assuring continuous high quality of care for their patients. Since the early Eighties a number of studies and reviews have demonstrated the positive influence of continuing medical education (CME) as well as continuing professional development (CPD) on physicians' knowledge and competence [2,3]. Properly planned and well designed learning activities (e.g. interactivity, use of multiple methods, multimedia CME, case-based learning, and multiple exposures) have been shown to even change physicians behaviour [3,4]. Some previous studies have suggested the types of media preferred by general practitioners and hospital doctors specially in Germany for their learning activities, including aspects about new media [5-7].

In the field of Complementary and Alternative Medicine (CAM) some articles have described the use of different kinds of new media for CME. Arlt et al. evaluated an E-Learning module for naturopathy in veterinary medicine which was appraised as a reasonable complement“ by the attending students [8]. Similarly, Peacock and Hooper showed the potential of E-Learning in the field of physiotherapy [9].

One of the most popular and specific therapeutic approaches of CAM in Germany is homeopathy provided by both physicians but also healing practitioners. German physicians practicing homeopathy are required to hold a special qualification in order to be eligible for reimbursement [10]. Healing practitioners in Germany are licenced by the state. Within the last years certification systems have increasingly been established to foster the education and qualifications of healing practitioners [11]. However, training and practice varies as there are different organisations offering such courses. As a consequence the market for homeopathic education has increasingly diversified with old (books, seminars) and new media (video-seminars, pc-programs, homeo-wiki and internet-courses).

However, very little is yet known about homeopaths’ preferences in using educational media and their requirements of this topic. Only one paper of Oettmeier et al. [12] reported on the use of interactive learning media in homeopathic further education. This survey was designed to gain a better understanding of the usage, requirements and appraisal of educational media by homeopaths.

## Methods

At an educational conference on homeopathy in 2009 with a focus on video case reports on Sankaran’s Sensation method participants (physicians and healing practitioners) were asked to participate in this survey.

The Sensation Method, developed by Raja Sankaran and colleagues, is an addition to classical homeopathic case taking focusing on coherent patterns of experience in the patients' presentation of complaints. Sensations are conveyed by groups of words (for instance “pressure”, “constriction” and “heavy”), repeatedly used by the patient to describe different symptoms regarding the body, emotions and mind. The exact choice of words and the patients’ use of hand gestures are essential for Sensation Method case taking. Seminars on Sensation Method therefore often use video case presentations for illustration [13,14].

The questionnaire consists of 53 items covering the topics “homeopathic practise” (24 items), “formal education and context of work” (9 items), “utilization of educational media” (9 items) and “favoured attributes for educational media” (11 items).

All questions regarding the “homeopathic practise” were to be answered on a 5-point Likert-scaled from never to always (0 = never; 1 = seldom; 2 = sometimes; 3 = often; 4 = always) except of two questions on the personal history of homeopathic practice and its’ proportion in daily work. The questions on “formal education and context of work” were provided on nominal scales and in case of specification (i.e. additional qualifications) free text fields were available. The items “utilization of educational media” (9 three-point Likert scaled items with 0 = never, 1 = sometimes, 2 = often) and “favoured attributes for educational media” (11 three-point Likert scaled items with 0 = unimportant, 1 = more or less important, 2 = very important) were adopted and modified from an already existing questionnaire by Vollmar et al. [15].

## Ethical approval

As this was a non-interventional survey which did not include human information or material and participation in the survey did not affect patients' treatment in any way no ethical approval was required.

### Statistical analysis

Apart from descriptive statistics providing percentages, means, standard deviations, and median, explorative statistical analysis was used to detect

A.) differences between physicians and healing practitioners within the sample.

B.) differences in mean values of items compared to the study on the use of educational media in German general practitioners given in Vollmar et al. [15].

To test for group differences we used Chi-Square statistics in case of nominal or ordinal variables and Wilcoxon rank-test for ordinal variables. Similar to the approach of Vollmar et al. [15] we provided frequencies and percentages. Furthermore, we used the Chi-Square statistics for independent samples to detect differences between homeopaths and conventional GPs. Independent of the statistical test we always judged a difference to be significant at p < 0.05. We used SPSS 15.0 to calculate the tests of our survey and the Java based Online Applet JUMBO [16] to calculate Chi-Square test statistics for given proportions.

## Results

Out of 192 conference attendants 118 participated in the survey (61.5%) consisting of n = 80 (69%) physicians and n = 36 (31%) health practitioners (HP). The mean age was 49 years (SD 7.5 years). The participants reported a mean of 12 years of homeopathic practice (SD 6.4) and the use of homeopathy in daily practice was 67.9%. 35% stated good knowledge and 42.5% moderate knowledge about the sensation method homeopathy according to Sankaran [13,14].

While differences in age (Mann–Whitney U-Test, p = 0.627), gender (Fisher exact test, p = 0.09), and years of homeopathic practice (Mann–Whitney U-Test, p = 0.114) were not significant between physicians and health practitioners, we found significant group differences with respect to proportion of homeopathy in daily practice (Mann–Whitney U-Test, p < 0.001) and years since approbation/HP-diploma (p < 0.001). Table 1 provides more detailed information on the socio-demographic data of the participants.

**Table 1:** Socio-demographic data of the survey participants

	**Physicians**	**Health practitioners**	**Total**
**N (%)**	81 (68.6%)	37 (31.4%)	
**Mean age**	49.5 ± 7.3 years	48.9 ± 8.0 years	49.3 ± 7.5 years
**Median age (min - max)**	49 (28-69)	49 (31-70)	49 (28-70)
**Gender (N /%)**	20 / 24.7% male	4 / 10.8% male	24 / 20.3% male
**Year since approbation or HP-diploma**			
**Mean Std.**	1987 ± 8	1998 ± 7	1990 ± 9
**Median (min - max)**	1986 (1974 – 2005)	1998,5 (1983-2007)	1989 (1974 – 2007)
**Years of Hom. Practice**			
**Mean Std.**	12.7 ± 7.0	6.5 ± 6.4	12.0 ± 6.8
**Median (min - max)**	11 (2-30)	10 (2-25)	10 (2-30)
**% of Hom. in Patient Care**			
**Mean Std.**	59.3 ± 34.2%	86.6 ± 18.9%	67.9 ± 32.7%
**Median (min - max)**	65% (10-100%)	90% (30-100%)	80% (10-100%)

**Figure 1:**
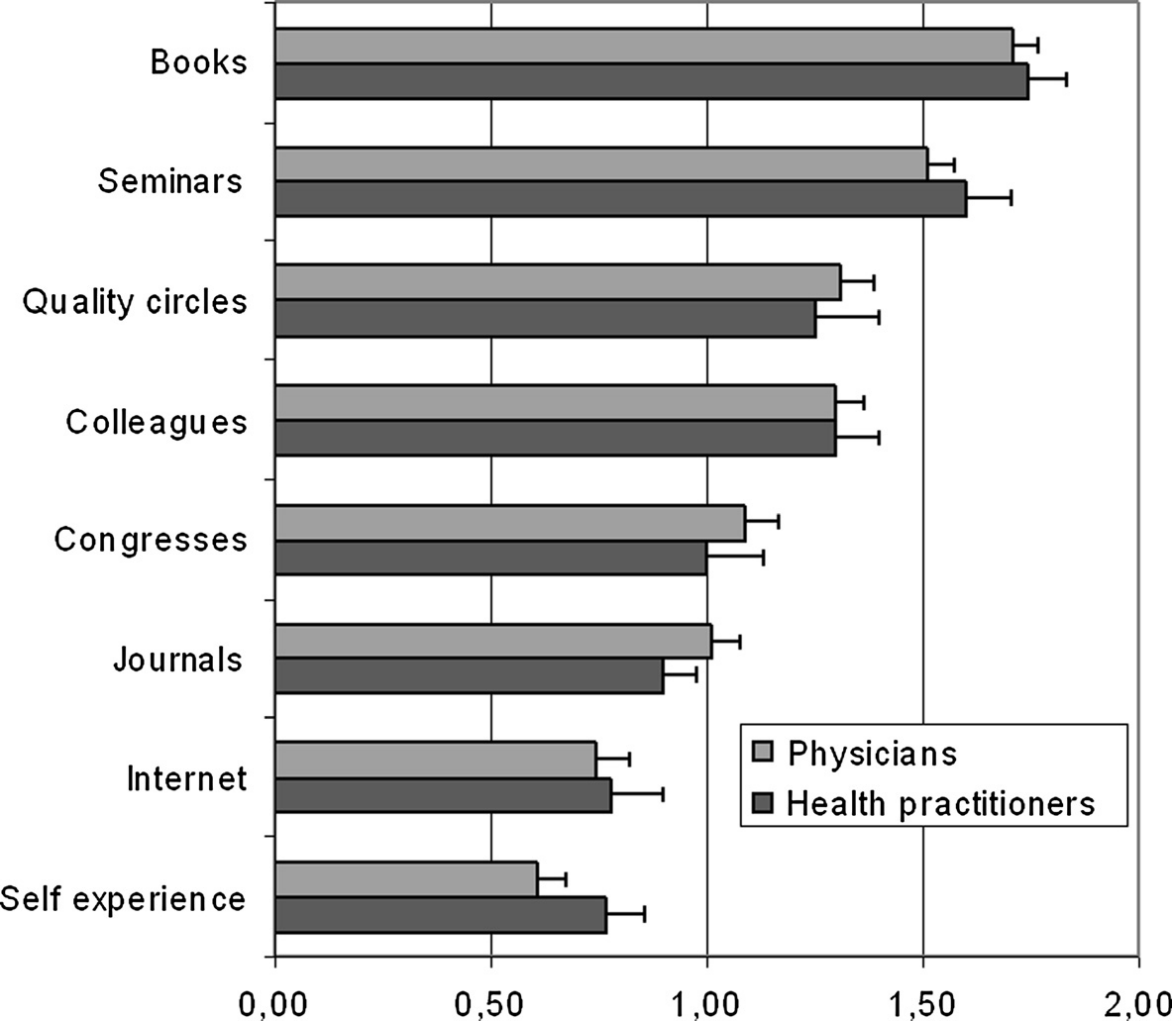
**Use of media for Homeopaths’ continuing medical education subdivided by profession. **(Scale values: 0 = never, 1 = sometimes, 2 = often).

For their continuing homeopathic education survey participants predominantly indicated to use books (1.72 ± 0.49) and seminars (1.54 ± 0.54). Moderately favored were colleagues (1.30 ± 0.57), quality circles (1.29 ± 0.73) and congresses (1.07 ± 0.66). Quality circles are regular regional meetings of GPs to discuss clinical topics, guidelines, and other ways to improve the quality of care as well as new developments in politics and funding. The participation of German GPs in QCs is mandatory in order to be part of most of governmentally funded disease management programs. Less favored were scientific journals (0.98 ± 0.51), the internet and E-learning (0.76 ± 0.62) or self experience (0.65 ± 0.57) (see details in Figure 1). Statistical analysis of the differences between professions using the Mann-Whitney-U test did not find significant differences. The most highly favored attributes concerning medical education media were reliability (1.76 ± 0.49), relevance for clinical practice (1.74 ± 0.54) and user friendliness (1.60 0.57). Moderately favored were the attributes concise (1.38 ± 0.60), case-related (1.36 ± 0.67) and fast (1.27 ± 0.68). Less favored were inexpensiveness (1.10 ± 0.53), graphical material (0.92 0.61) and interactivity (0.88 ± 0.66). Again, the statistical analysis of the differences between professions did not find any significant differences between the professions except for the item “inexpensiveness”.

Differences in the subgroups of our sample were small. We therefore decided to pool both groups in the comparison with general practitioners from an earlier survey [15].

### Comparison with GPs from earlier research

When comparing basic demographic data to general practitioners in earlier research [15], our sample was similar in age (51.1 ± 7.1 vs. 49.3 ± 7.5) but differed in gender (female 28.5% vs. 75.3%), profession (100% MD vs. 68.6% MD and 31.4% HP) and year of last examination at university or HP-diploma 1983 ± 7 vs. 1990 ± 9. In contrast to the homeopaths (physicians and health practitioners) of our survey GPs from the former study used the following educational media more often: quality circles (75.7% vs. 45.6%, p < 0.001), scientific journals (64.1% vs. 12.1%, p < 0.001), colleagues (59% vs. 35.6% p < 0.001) and with less frequency conferences or congresses (38.5 vs. 25.2%, p < 0.001). The internet and e-learning were used more often by GPs than homeopaths (20.5% vs. 9.6%), yet this trend was not statistical significant (p = 0.054). The homeopaths only used books more often than the general practitioners (73.8% vs. 39.7%, p < 0.001). All details are provided in Table 2.

**Table 2:** Differences between conventional GPs and Homeopaths in utilization of preferred educational media

** *How often do you use the following educational media? * **						
		**Never**	**Sometimes**	**Often**	**Chi-Square**	**p-Value**
*Colleagues*	*GP*	*3 (1.1%)*	*104 (39.8%)*	*154 (59%)*	20.055	< 0.001
	Hom	6 (5.8%)	61 (58.7%)	37 (35.6%)		
Quality circles	*GP*	*2 (0.8%)*	*62 (23.6%)*	*199 (75.7%)*	50.752	< 0.001
	Hom	17 (16.5%)	39 (37.9%)	47 (45.6%)		
*Books*	GP	7 (2.7%)	151 (57.6%)	104 (39.7%)	*35.652*	*< 0.001*
	Hom	2 (1.9%)	26, (24.3%)	79 (73.8%)		
*Conferences & Congresses*	GP	10 (3.8%)	151 (57.6%)	101 (38.5%)	*23.702*	*< 0.001*
	Hom	19 (18.4%)	58 (56.3%)	26 (25.2%)		
*Scientific Journals*	GP	2 (0.8%)	91 (35.1%)	166 (64.1%)	*94.788*	*< 0.001*
	Hom	15 (14.0%)	79 (73.8%)	13 (12.1%)		
Internet & E-Learning	*GP*	*83 (32%)*	*123 (47.5%)*	*53 (20.5%)*	5.834	0.054
	Hom	32 (34.0%)	53 (56.4%)	9 (9.6%)		

Although GPs’ and homeopaths’ requirements for preferred educational media were valued similarly, GPs were more affirmative to ‘relevance to practice’ (93.3% vs. 78.3%, p < 0.001) and ‘scientific reliability’ (90.9% vs. 79.0%, p < 0.001). Both preferred ‘user-friendliness’ (68.1% vs. 64.1% p = 0.756) and to some extend ‘fast’ (58.4% vs. 39.6%, p = 0.0017). ‘Concise‘ was clearly more preferred by GPs than it was by homeopaths (71.8% vs. 44.1%, p < 0.001). ‘German language’ was of less importance for homeopaths than for GPs (19.8% vs. 9.9%, p = 0.014). Similarly, the remaining items were important only to a few participants in both samples: ‘interactive’ (16.7% vs. 15%, p = 0.852) and ‘with graphical material’ (17.3% vs. 14.9%, p = 0.829). All details are provided in Table 3.

**Table 3:** Differences between conventional GPs and Homeopaths in requirements for preferred educational media

** *How important do you rate the following requirements in medical information media?* **						
		**Unimportant**	**Less important**	**Very important**	**Chi-Square**	**p-value**
*Fast*	*GP*	*13 (5.2%)*	*91 (36.4%)*	*146 (58.4%)*	12.759	0.0017
	Hom	13 (12.9%)	48 (47.5%)	40 (39.6%)		
*Reliable / scientific*	GP	0	23 (9.1%)	229 (90.9%)	*13.453*	< 0.001
	Hom	3 (2.9%)	19 (18.1%)	83 (79.0%)		
*Concise*	GP	0	71 (28.2%)	181 (71.8%)	*33.591*	< 0.001
	Hom	6 (5.9%)	51 (50.0%)	45 (44.1%)		
Relevant to practice	*GP*	*0*	*17 (6.7%)*	*236 (93.3%)*	21.889	< 0.001
	Hom	5 (4.7%)	18 (17.0%	83 (78.3%)		
With graphical material	*GP*	*59 (23.2%)*	*151 (59.4%)*	*44 (17.3%)*	0.375	0.829
	Hom	23 (22.8%)	63 (62.4%)	15 (14.9%)		
German language	*GP*	*25 (9.9%)*	*125 (49.4%)*	*103 (40.7%)*	8.48	0.014
	Hom	21 (19.8)	39 (36.8%)	46 (43.4%)		
Interactive	*GP*	79 (31.1%)	137 (53.9%)	38 (15%)	0.321	0.852
	Hom	29 (28.4%)	56 (54.9%)	17 (16.7%)		
User friendly	*GP*	8 (3.2%)	72 (28.7%)	171 (68.1%)	0.56	0.756
	Hom	4 (3.9%)	33 (32.0%)	66 (64.1%)		
Cost-effective	*GP*	30 (12%)	130 (51.8%)	91 (36.3%)	10.76	0.005
	Hom	9 (9.4%)	68 (70.8%)	19 (19.8%		

## Discussion

For the first time this article gives insights into preferences and appraisal of educational media of homeopaths. Although our sample consisted of physicians and health practitioners, both subgroups tended to vote similarly with respect to their media preferences and were compared to a sample of GPs in Germany. For their continuing education homeopaths predominantly indicated to use books and seminars whereas journals and the internet were used less. The most favoured attributes concerning medical education media were reliability, relevance for clinical practice and user friendliness. Less favoured were inexpensiveness, graphical material, and also interactivity.

These results might be surprising with respect to computer literacy. Homeopathic repertories and materia medicae have been converted into computer programs within the last 30 years using modern database technology and search engines [17]. Thus, practical day-to-day work of homeopathic therapists includes working in a modern interactive information technology environment. Nevertheless, this does not seem to alter the habits when it comes to CME. However, as this was the first study in the field of homeopathy this effect should not be overestimated.

Since this survey was conducted, several new online based educational courses have been offered for homeopathic practitioners. Future surveys might therefore come to different results regarding the use of the internet for CME in homeopaths.

Because of the lack of surveys in the field of CAM respectively homeopathy we compared our results with a study sample of 264 GPs from an earlier study [15]. In that study GPs favored learning environments such as journals, colleagues, and quality circles. Similarly, new media like the internet was used less often for their learning activities, even though the use of the internet in general was quite high. The most important requirements for media in medical education as perceived by the participants in our study were its ‘relevance for daily practice’ and ‘reliability’ which is in accordance with the findings of Vollmar et al. [15]. Moreover, this also reflects the requirements on electronic media given by homeopathic experts [18].

**Figure 2:**
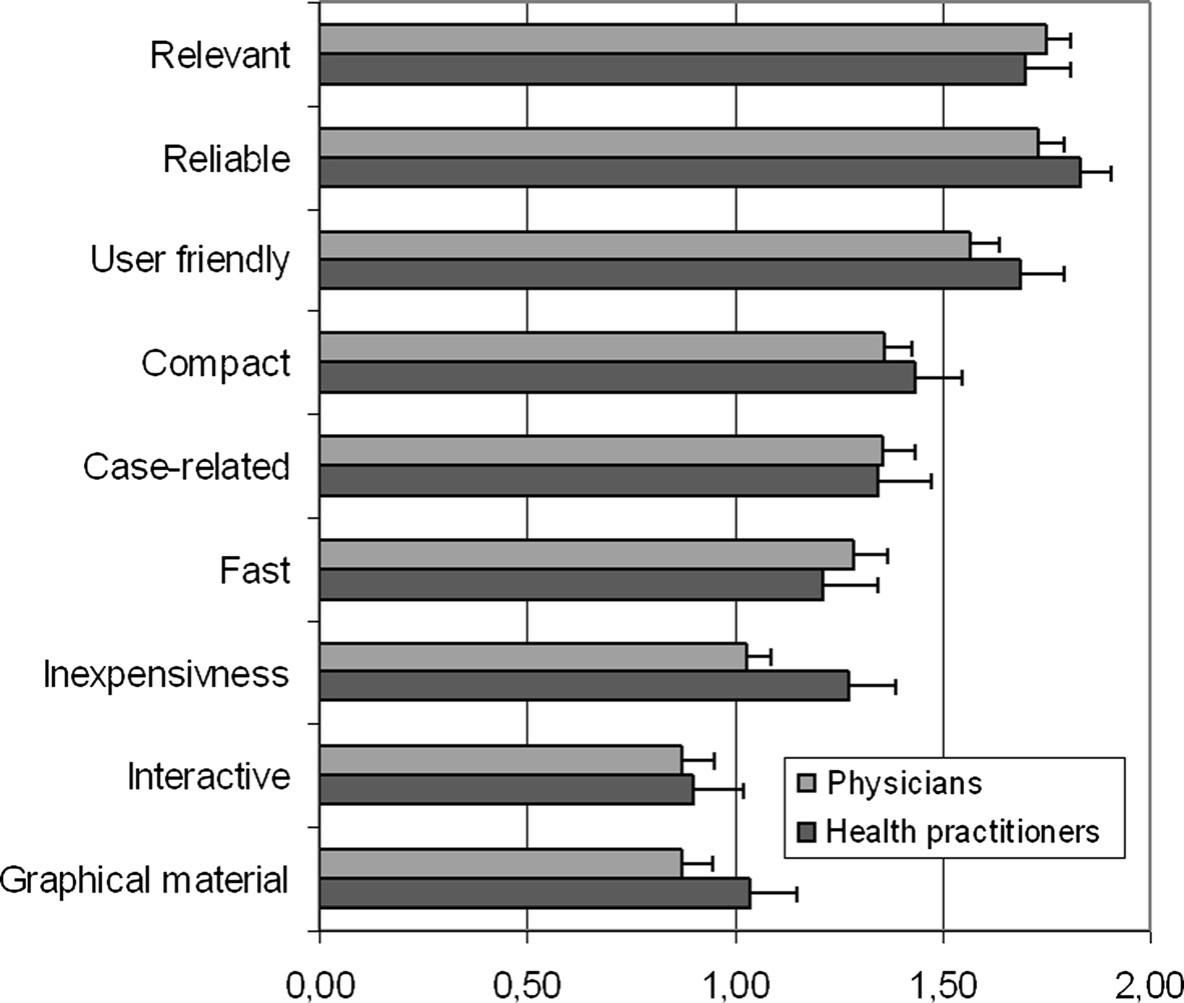
**Most favoured attributes concerning medical education media subdivided by profession. **(Scale values: 0 = unimportant, 1 = more or less important, 2 = very important).

Interestingly, we found significant differences between a previous sample and our survey population [15]. Some differences like the use of quality circles may be due to the fact that the study of Vollmar et al. was conducted in GPs organized in QC and thus it could be regarded as a comparison bias. Contrary to expectations, homeopaths visiting an educational conference on homeopathy with a focus on video case reports stated graphical material mostly as “less important” and even slightly less than conventional GPs. Furthermore, case-relatedness was the 5^th^ important item of most favoured attributes concerning CME media (Figure 2).

The most obvious and intriguing discrepancy was found in the use of books for CME. While homeopaths ranked books the highest for their learning activities they were only mid-fielder in GPs ranking. This may be explained by a relatively wide core of traditional knowledge of homeopathy which also today is still published in books. In our special case the fact that most of the participants are using Sankaran’s method which is mainly published in books might also explain the high relevance of books for this sample. Moreover homeopathic drug proving have no expiry date. This implies that homeopathic knowledge has a more cumulative character and short-term updates are not that necessary as in pharmacological databases.

Last but not least due to the public domain character of homeopathical standard literature many unreliable translations haunt the internet and thus it might not be surprising that there is a preference for books from a trustworthy publisher.

Thus, homeopathic books (repertories, materia medicae and compendia) can still be regarded as the most important source of valuable knowledge for daily practice. According to a recent representative survey from the German Association of Homeopathic physicians 57% of homeopathic GPs stated to use a printed homeopathic repertory and 88% of those who used an electronic repertory program ‘often’ or ‘always’ verified the results in a printed material medica in case of chronic and 57% in the case of acute diseases [19]. This might also account for the complete spectrum of CAM information. A study by Dooley et al. showed that Australian oncology practitioners were found to mostly use paper-based materials such as textbooks and journals on CAM [20].

According to Kösters [19], 77% of the homeopathic GPs use an electronic repertory program to specify the homeopathic remedy. Nevertheless, the internet still does not seem to be the medium for learning activites. In accordance to the results by Vollmar et al. the retrieval of information through the internet is not recognized as a learning activity [15]. This is supported by the survey of the German Association of Homeopathic physicians in which only 24% of the homeopathic GPs stated to use the internet to gain information [19].

The results are somewhat surprising, as a current review revealed a number of 45 online databases providing a broad variety of CAM information with 4 bibliographic databases on homeopathy ranging from clinical to basic research [21]. The application of such new information technologies in CME is still recommended in order to have a lasting impact on the physicians' working environment and their learning behaviours [9,22].

This is also underpinned by the Final report of the European Union on Information and Communication Technology (ICT), which concludes that most importantly “European GPs would prefer if the issue of eHealth were included in the curricula of medical education” [23].

A lack of ICT training for GPs as the most probable and strongest hindering factor for using eHealth applications identified in that survey can be ruled out in our case, as homeopaths are quite familiar with electronic media due to electronic repertorisation. Solutions for electronic learning environments which have a higher degree of synergy between traditional and new media, i.e. by using innovative retrieval technology and app-technology might be future directions which might help to overcome the barrier between classic and new media [24].

### Limitations

The results of our study are based on a convenience sample of homeopaths attending a learning conference and cannot be regarded as representative for homeopaths in Germany in general. According to the survey summarized by Kösters [19], only 26% of the homeopathic GPs are using the method of Sankaran/Mangialavori, on which the conference topic was based. Thus, GPs and healing practitioners practising according to another homeopathic school might vote differently according to the use and appraisal of educational media.

In our survey the response rate was quite good with 61.5%, although a bias based on respondents cannot be ruled out as well as it could be a bias for the preference of conferences.

The comparison of homeopaths with the general practitioners from a former survey regarding preference of educational media and their requirements [15] has the following limitations: Firstly, baseline data differ in gender and profession and thus are only limited comparable. Secondly, the GPs in the comparison study were recruited as participants of quality circles. Thus, a selection bias has probably influenced at least one question regarding quality circles. Finally, there is no information to what extend the GPs of the comparison group are practising homeopathy or CAM. Thus the difference might have been in more extreme in a sample of purely conventional GPs. Nevertheless, other studies with different and partly representative samples of GPs sustain these results [5,7,15,25].

## Conclusion

For CAM respectively homeopathy quality assuring activities including formalized educational programs have increased. Although CME in Germany is still behind other international trends it was rendered mandatory for GPs in Germany in January 2004 [6,8,15]. Our results illustrate the situation of medical education media in homeopathy in Germany and may contribute to CAM-related CME/CPD development strategies. Further investigations are indicated to find optimal learning media environments especially in the field of CAM.

## Competing interests

The authors declare that they have no competing interests.

## Authors’ contribution

ME designed the study, participated in the acquisition of data, and drafted the manuscript. TO made substantial contributions to the interpretation of data and performed the statistical analysis. HCV helped with the interpretation of the data in the context of family practice and in drafting and critical revising of the manuscript. AH participated in design and coordination of the study and organised the process of data management. CR contributed homeopathic aspects of the study, literature search and in interpreting the results of the study. All authors read and approved the final manuscript.

## Pre-publication history

The pre-publication history for this paper can be accessed here:

http://www.biomedcentral.com/1472-6882/12/95/prepub
